# Having a Say in Research Directions: The Role of Community Researchers in Participatory Research with Communities of Refugee and Migrant Background

**DOI:** 10.3390/ijerph19084844

**Published:** 2022-04-15

**Authors:** Fran Hearn, Laura Biggs, Stephanie Brown, Lien Tran, Sherinald Shwe, Ta Mwe Paw Noe, Shadow Toke, May Alqas Alias, Maryaan Essa, Shogoufa Hydari, Josef Szwarc, Elisha Riggs

**Affiliations:** 1 Murdoch Children’s Research Institute, Intergenerational Health, Melbourne 3052, Australia; 2 Department of General Practice, The University of Melbourne, Melbourne 3052, Australia; 3 Department of Paediatrics, The University of Melbourne, Melbourne 3052, Australia; 4 South Australian Health and Medical Research Institute, Adelaide 5001, Australia; 5 Department of Medicine, The University of Melbourne, Melbourne 3052, Australia; 6 WHO Collaborating Centre for Viral Hepatitis, The Doherty Institute, Melbourne 3000, Australia; 7 The Victorian Foundation for Survivors of Torture Inc., Brunswick 3056, Australia

**Keywords:** community researcher, trauma-informed research, cross cultural research, refugee health, migrant health, decolonising methodology

## Abstract

Research teams in high-income countries often fail to acknowledge the capacity and contributions of Community Researchers. This qualitative exploratory study used decolonising methodology and the Foundation House ‘Refugee Recovery Framework’ to understand Community Researchers’ perceptions and experiences of their role, and how research teams can integrate the knowledge they bring into research. Purposive sampling was used to facilitate the recruitment of eight Community Researchers from five different community groups working in Melbourne, Victoria. Semi-structured interviews lasting forty to sixty minutes occurred between December 2020 and January 2021. Data were analysed using reflexive thematic analysis. Findings reported in this paper include eight themes: ‘nothing about us without us’; ‘open the door’; a safe space to share; every step of the way; this does not translate; finding the right way to ask; a trauma-informed approach; and support within the workplace. The knowledge obtained demonstrates that Community Researchers facilitate meaningful participation in research for women, families, and communities of refugee or migrant background. Community Researchers’ presence, knowledge, and skills are vital in establishing culturally safe research practices and developing accessible language to facilitate conversations about sensitive research topics across multiple languages. Community Researchers can make important contributions at all stages of research, including data collection and interpretation.

## 1. Introduction

### 1.1. Research with Women, Families, and Communities of Refugee and Migrant Background

In 2019, one in three women giving birth in Australia were born overseas, with the majority re-locating from non-English speaking countries [1]. The latest available census data shows that 73% of people who migrated to Australia with a permanent visa speak a language other than English at home, and this number increases to 94% for those with a humanitarian visa [2]. Women, families, and communities who are unable to speak or read English are often excluded from perinatal research due to the practical, methodological, and ethical complexities associated with working in a cross-cultural or multi-language context [3,4,5,6]. Other barriers to participation include culturally unsafe, inappropriate, or insensitive research practices that can feel disrespectful [3,4,6,7]. Facilitating meaningful access to research participation is critical to ensuring that research findings are relevant to the social and cultural context of refugee and migrant background communities [3,6,8].

### 1.2. Inequities during the Perinatal Period

In Australia and other high-income countries, women of refugee and migrant background experience a greater burden of adverse perinatal outcomes, including higher rates of stillbirth, preterm birth, caesarean birth, congenital anomality, admission to special care or neonatal intensive care, and trauma-related mental health concerns [9,10]. Maternal physical and mental health and child health outcomes can be affected by parents’ prearrival and settlement experiences, which can involve the loss of family, friends, and community; social isolation; adapting to a new culture and language; and discrimination [9,11,12]. Women of refugee background may also experience additional layers of hardship that can affect health outcomes, such as displacement, torture, and food insecurity [13,14,15].

Despite the availability of publicly-funded maternity care in Australia and many other high-income countries, culturally unsafe systems and approaches to care; inadequate access to interpreters; inadequate support to access transport and attend appointments; mistrust of services or authorities; and lack of familiarity with local health care systems perpetuate inequities [9,12,14,15,16,17]. This combination of factors affects women’s and families’ capacity to engage with health care, and to understand and integrate important information relevant to health and wellbeing during pregnancy [9,12,18,19]. Building a strong understanding of the experiences of families of refugee and migrant background accessing maternity and early childhood health services is needed to underpin evidence-based approaches to the provision of culturally safe and responsive health care [8,9,10,12,16].

### 1.3. Thinking Differently about Research Practices

The majority of research undertaken by our team involves women, families, and communities of refugee or migrant background during the perinatal period. Engaging families of refugee or migrant background in research requires researchers to think differently about research methodologies, methods, and approaches. In 2011, our research group undertook a ‘proof of concept’ study working in partnership with the Victorian Foundation for Survivors of Torture (Foundation House) to adapt and test research practices for engaging Afghan families in a research project about their experiences of maternity and early childhood health services in the South East of Melbourne [20]. Foundation House is a non-denominational, non-government, and not-for-profit organisation that receives funding from the Victorian and Commonwealth Governments, charitable funds, and donations. Their work provides specialist support services to people of refugee background [14]. In this project, we established a governance structure that gave equal weight to the knowledge and expertise of researchers, and to the knowledge and expertise of Foundation House staff, leveraging the strength of their connections with refugee communities in Victoria. We collaborated to write the study protocol and oversee the conduct of the study.

One of our first steps involved the appointment of two ‘Community Researchers’ from within the Afghan community, to facilitate community consultation and engagement in the research. The research methods used in the Afghan Families Study were developed collaboratively with input from Community Advisory Groups and other community members consulted about the research, and from the Community Researchers themselves. The two Community Researchers appointed to work on this study worked with other members of the research team to facilitate community engagement processes in preferred languages; provided early and ongoing guidance on community priorities, project relevance, and the suitability of research methods; and contributed to data collection, translation and transcription of discussion groups and interviews; and interpretation and dissemination of study findings [20]. These responsibilities have since remained a foundation of the role.

The Afghan Families Study was underpinned by the recognition of inherent power imbalances in research settings and a corresponding commitment to continuous, reciprocal knowledge exchange between research team members and with community partners. Foundation House—as a community partner working in refugee health and advocacy—brought a deep understanding of the ‘refugee experience’ to the partnership and a conceptual framework for working with refugee communities. The Foundation House ‘Refugee Recovery Framework’ foregrounds a recognition of the human rights violations and persecution experienced by people of refugee background, the psychological impact of these experiences, the way in which these manifest in trauma reactions, and goals for healing and recovery [14]. All research team members involved in the Afghan Families Study participated in training facilitated by Foundation House on trauma-informed principles and the ‘Refugee Recovery Framework’. A major element of co-designing the study involved the development of research approaches that applied this framework. Importantly, this included the development of approaches recognising the potential of research processes—for example, interview methods or topics covered in discussion groups and interviews—to be re-traumatising. The Community Researchers’ advice and engagement with the Afghan community with regard to co-design and implementation of research procedures, such as informed consent procedures and interview methods, was critical to the team’s capacity to embed a trauma-informed approach consistent with the Foundation House ‘Refugee Recovery Framework’.

This approach has now been adapted and implemented in a series of studies conducted by our research team in partnership with Foundation House over the past decade [16,21,22,23,24,25]. During this time, we have worked with Community Researchers with diverse cultural and linguistic backgrounds matched to the communities we have sought to engage in our research and expanded our scope to include migrant communities. We choose to use the term ‘community’ in an inclusive sense, whilst acknowledging that community groups are not homogenous. Although Community Researchers are employed to work broadly within their own communities, subcultural diversity means that some of those we seek to work with may not necessarily share the same cultural identity or language as the Community Researcher. We are not always able to reach all subcultural groups within a community. The knowledge and connections that Community Researchers have brought to our team have been critical to the quality and integrity of the work we have undertaken together. The inclusion of Community Researchers in our research team has strengthened our capacity to engage meaningfully with culturally diverse communities, promote social inclusion, and build individual and community capacity. Importantly, it has also strengthened our capacity to explore insights that may otherwise have been lost in translation, interpret community experiences and perspectives, and confirm research findings. Working with Community Researchers has challenged us to think deeply about what it means to create and maintain a culturally safe team environment that values cultural diversity and recognises the potential for research practices to be re-traumatising, and the inherent power imbalances that exist in research settings. Unfortunately, funding for the Community Researcher role has historically been received per project rather than on a secure and ongoing basis. Precarious funding has impacted on the availability of secure employment pathways, and Community Researchers are often employed on a casual basis.

In this paper, we report findings from a qualitative exploratory study in which we asked Community Researchers to reflect on their experiences of working in our team. The specific aims of the study were to: (i) explore how Community Researchers understand their role; (ii) investigate Community Researchers’ experiences of doing this work; and (iii) understand from the perspective of Community Researchers what it takes to integrate the knowledge they bring into different stages of the research process.

## 2. Materials and Methods

### 2.1. Methodology

This qualitative exploratory study was theoretically underpinned by a decolonising methodology, which acknowledges the historical and ongoing impacts of imperialism, colonialism, racism, and other forms of discrimination and injustice [26]. Although practical application can vary, decolonising approaches to research re-centre the perspectives, knowledge, and wisdom of populations or communities who experience these forms of oppression [26,27,28]. The decolonising intent within this study included a commitment to reciprocal knowledge exchange and the incorporation of trauma-informed principles congruent with the Foundation House ‘Refugee Recovery Framework’.

### 2.2. Eligibility and Recruitment

Eligible participants included all Community Researchers employed by the Refugee and Migrant Research Program at the Murdoch Children’s Research Institute (MCRI) between 2014 and 2021. Careful consideration and planning were undertaken to ensure that Community Researchers approached to take part did not feel compelled to participate. For example, direct line managers were not involved in recruitment or conducting interviews. Participant information emphasised the Community Researchers’ right to decline to participate without any consequences for their future employment. Study procedures were developed to ensure that the decision of any Community Researcher not to participate would remain confidential. A staff member (FH), who was not involved in supervision of Community Researchers, initially approached prospective participants via email. Each individually tailored email included a secure hyperlink to a web portal, Research Electronic Data Capture (REDCap) [29], where participants could express their interest in completing an interview and nominate important details, such as preferred mode of contact. Participants were offered the choice of completing an interview with any one of three staff members (FH, ER, SB), none of whom were involved in direct supervision. Prior to each interview, informed e-consent was obtained using REDCap [29]. Upon completion of the interview, participants were provided with a thirty-dollar supermarket gift voucher as a token of appreciation for their time.

### 2.3. Ethics and Consent

Ethics approval was obtained from the Royal Children’s Hospital (RCH) Human Research Ethics Committee (HREC). Critical issues that were planned for included: ensuring consent for participation was voluntary, guaranteeing that participation would not impact employment, protecting privacy and confidentiality, considering emotional impacts for participants and the researcher, secure data management, and implementation of a COVID-19 safety plan.

### 2.4. Data Collection

Data were collected via semi-structured interviews [30], conducted in English between December 2020 and January 2021, over the telephone or by Zoom video software [31], as per participant preference. Remote data collection was necessary due to COVID-19 directives in force at the time that prohibited face-to-face contact for non-essential work. An interview schedule was utilised to guide and contain each conversation, although iterative and spontaneous lines of questioning also occurred to contemporaneously incorporate the researcher’s understandings as the study progressed [30]. The interview schedule was piloted with one person prior to use. No changes to the interview guide were recommended. To begin each interview, participants were asked to describe what they did in their Community Researcher role. Then they were asked to reflect on their experiences of field work such as recruitment, data collection, transcription, translation, and community engagement. The interviews lasted for forty to sixty minutes, were audio-recorded with consent, and professionally transcribed for analysis. Transcripts were checked for accuracy and anonymised by the interviewer, meaning that names of people, community groups, services, and locations were removed to protect participant identity (FH).

### 2.5. Saturation

Although sometimes used as a measure of quality in narrative-based research [32], this study did not attempt to gauge data saturation due to the finite number of participants available for recruitment [33]. Purposive sampling within a known cohort ensured that every participant was able to provide highly specific and relevant data [33]. Thematic analysis was approached using a reflexive technique, meaning that codes and themes continued to evolve in response to the increased interpretations and understandings of the researcher [34]. As such, our approach demonstrates differing measures of quality, which will be extrapolated below within the subsection ‘Trustworthiness’.

### 2.6. Trustworthiness

Trustworthiness is a term used to encompass measures of significance and value in qualitative research [35,36]. This study achieved trustworthiness in a variety of ways. Data were securely managed to remain audit-ready using appropriate software. Data analysis was systematic in approach, including iterative coding, categorisation, and theme development. Ongoing team discussion and evaluation of the evolving analysis occurred to ensure an appropriate level of interpretation was applied to the data when developing themes. A reflective journal was kept by the first author and interviewer, including contemporaneous notetaking of impressions following each interview. Researcher conduct and positionality was determined by decolonising methodological views, and informed by the Foundation House ‘Refugee Recovery Framework’ [14]. A further description of the framework’s practical application and congruence with decolonising methodology will be provided below within the subsection ‘Reflexivity’.

### 2.7. Reflexivity

All authors have engaged with the ‘Refugee Recovery Framework’ [14], which requires thoughtful consideration of potential trauma-related impacts for research participants, and facilitates the integration of a trauma-informed approach into research. Participants are considered likely to have either personally experienced or been impacted by trauma; disclosure of trauma is not required for researchers to interact safely and with due precaution [37]. Researchers aim to operate with a high level of professional accountability to participants, communities, and partner organisations. The framework supports researchers to understand the interplay of cultural, cross-cultural, historic, social, and political factors that impact upon experiences of resilience, healing, and recovery for individuals and communities of refugee background [14]. In congruence with decolonising methodology, the framework also acknowledges historic and ongoing impacts of imperialism, colonialism, and racism for individuals and communities of refugee and migrant background [14]. Critically reflexive exercises were systematically defined within the study protocol, with an aim to increase awareness of beliefs, assumptions, and power dynamics [28]. Throughout each phase of the research project, the first author (F.H.) maintained a reflexive practice via regular supervision and journaling. Whole of team activities included ongoing attendance at project meetings, group reflection, and contemporaneous feedback processes to progress thinking, analysis, and writing.

### 2.8. Data Analysis

Braun and Clark’s [38] method of reflexive thematic analysis was used to identify and interpret patterns of meaning within the data [39,40]. To ensure immersion, transcripts were read by the interviewer (F.H.) multiple times while listening to the audio recordings [41]. Transcripts were coded (F.H.) with descriptive labels reflective of meaning and context, using NVivo qualitative data software [42]. After one complete cycle of coding, all codes and categories were documented, presented, and discussed as a team (F.H. E.R., L.B.). Data were then uncategorised, re-coded, re-categorised, meaning checked, and corroborated (F.H., L.B.). New codes and categories were identified during this iterative process. Findings of codes and categories were visually mapped using two different figures to enhance understanding of categorical relationships, and critically discussed as a group (F.H., L.B., S.B.). Themes were extracted from the data through the combined analytical efforts of coding, categorising, critical reflection, and team discussion, whilst keeping the aims of the paper in mind [40,41,43,44]. Findings were then drafted collaboratively via writing, re-writing, reflection, and discussion (F.H., L.B., S.B., L.T., S.S., T.M.P.N., S.T., M.A.A., M.E., S.H., J.S., E.R.).

### 2.9. Participant Demographics

A total of eight eligible Community Researchers from five different community groups were approached for this study, and all agreed to participate. Professional background varied, encompassing a range of health and social care roles and qualifications. All participants held additional employment with other organisations, working across one or more professional roles that intersected with their Community Researcher role. Participants were all fluent in two or more languages, including English. Due to the small sample size, to prevent participant identity being deduced by readers, and to protect privacy, no further information regarding the demographics of study participants will be provided within this paper.

## 3. Results

**Figure 1:**
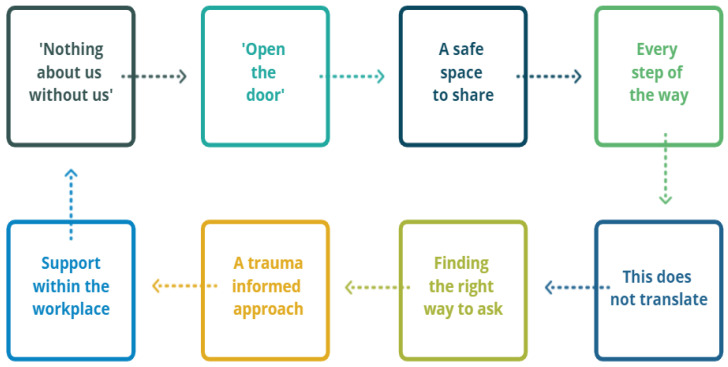
Interview themes: the role of Community Researchers in participatory research with communities of refugee and migrant background.

Community Researchers occupy a dual space physically, emotionally, metaphorically. The role is positioned inside their workplace, research team, and the individual research projects they work on, and at the same time, positioned inside their community. The duality of this positionality facilitates reciprocal understanding, respect, and trust between research and community. Community Researchers enable communities to build trust in research processes, and researchers to build understanding of communities. These findings are written with the voices of Community Researchers at the fore. Interpretations by the lead author (FH) aim to elucidate what was shared during interviews and re-centre important knowledge. Interview themes are listed below in Figure 1.

### 3.1. ‘Nothing about Us without Us’

Community Researchers perceive the shared cultural identity between themselves and their community as essential to the integrity of each project. Community Researchers emphasise the importance of self-determination and advocacy, “I always will make sure I stand for my community…”. (Participant 7); “It’s community-led…” (Participant 4); “I cannot talk enough about how important community is in terms of any intervention or any policy that involved them… ‘nothing about us without us’ is like my motto when I went to work with community.” (Participant 6). The way this role intersects with the right for communities to be self-determining is what makes our research relevant to Community Researchers, “… I felt like there is a need for it [the Community Researcher role] to do this project properly.” (Participant 4).

### 3.2. ‘Open the Door’

Community Researchers see themselves as the instigators or facilitators of a reciprocal respectful relationship between research organisations and community, “I think we really need someone from inside community to actually, to open the door…” (Participant 6); “I think for me, I get to build up trust with them and so, once I explain it to them, what the research will be about, they seem to be really interested and want to be a part of it and they want to contribute…” (Participant 8). When community members understand and value a project, they are more likely to engage and to share, “... you are just like a voice that they can hear, of course they are more willing to talk to you.” (Participant 7). Most of the communities that we involve in our research studies are new to research and experience inequitable access to health information and care, which can impact on the way a project is perceived or understood. Experiences of trauma, displacement, and settlement may also impact a person or a community’s capacity to engage; along with fear of authority, policing, governments, and the state, “… the research part is very new to the community as well so it was important for them to understand what this project is, and the research processes like audio-recording, getting all the questions right, so that it makes sense.” (Participant 4).

### 3.3. A Safe Space to Share

Often research conducted by our team involves topics that may be sensitive or bring up painful memories or experiences. For example, we have conducted research on maternal mental health during pregnancy, information given to women about stillbirth, and experiences of support for issues such as family violence. “I think they were really comfortable in sharing because I can feel that one woman shared about domestic violence and she told me that she never shared that with anyone before and I was feeling privileged, I was like, oh, I get to know everything about her and what she went through…” (Participant 8). Collecting data in languages other than English also means that we are working with people who are often ineligible to participate in other research, and therefore unable or unfamiliar with telling their stories through interview, “So, a lot of them might not even get the experience to tell their stories ‘cause some of them, there is no space…” (Participant 4). Community Researchers establish a physically, emotionally, and culturally safe space for participants to share, “… by the end of the interview everyone was saying, ‘I’m happy. You did a great job. It’s good to have someone who is from your community who understands your culture’.” (Participant 1).

Establishing and holding space to hear and acknowledge the stories of your own community feels significant. This aspect of the work is more than data collection. Community Researchers emphasise that increasing awareness and understanding of issues affecting their community promotes positive change, “Especially the women that are new here, so by the end of the interview each woman said, ‘You made us happy because we are feeling more comfortable now we know the [maternity] system, and we know that someone from our community who can talk our language. He [a doctor] can help us, we can go back and refer to him if we need something’. So, that was the most favourite part.” (Participant 1). Immersion in the stories can be personal, “Every time doing the interview, I was hearing new stories from women…” (Participant 1); “I think for me, working within my community, I get to find out so many issues that I didn’t know about before.” (Participant 3); “… they really opened up and shared a lot of things which I didn’t know about before, and I found really surprising, and I can see patterns in every interview.” (Participant 8). Responding to disclosures or distress can be rewarding and presents a timely opportunity to provide essential support or referral, “It felt good that the participants felt comfortable to share their stories and we were able to refer them to appropriate services.” (Participant 4); “… knowing their stories, giving them a chance to share it with someone and talk about their experiences, it was really enjoyable for me.” (Participant 5).

### 3.4. Every Step of the Way

Co-development of research studies with Community Researchers is collaborative and contemporaneously incorporates critical input and insight to promote cultural safety, “I enjoyed being part of the process… every step of the way being involved in it.” (Participant 4). For example, Community Researchers assist with the development and translation of interview questions, to ensure they are appropriate and make sense to their community, “I think it’s really good if they give their perspective on how we can be sensitive around some of the questions and what way is appropriate to do the interviews, I think it’s really important that the research can engage the community.” (Participant 3). Community Researchers also facilitate Community Advisory Groups, which seek to embed a variety of community perspectives within a project, to co-design structure and approach, “… I felt like I have the say in the project like the direction it should go and feeding back what the group and what is comfortable and what is appropriate.” (Participant 4). Community Researchers facilitate reciprocal communication across languages between the research team, community members and research participants, “…it’s hard to open to outsider people at first. But actually we [the community] really want to talk and if a bridge can be built then yes, they’ll talk a lot.” (Participant 5).

### 3.5. This Does Not Translate

Whilst unpacking the above aspect of the work in interviews, all Community Researchers expressed frustration with some of the challenges inherent to multilingual communication. Particularly when a word, a concept, or a feeling is untranslatable either verbally or in writing. That is, something exists in one language but not the other, “So, it’s hard because… you might not have the word in English and then if you’re trying to say a word you might have to translate to a few sentences to describe just one word.” (Participant 4); “Yeah, because if I write it in English, it will mean different thing.” (Participant 1); “… we don’t have a specific word for stillbirth so for me, it was really hard to just ask…” (Participant 8). Understanding supports confident translation or interpretation and more accurate, meaningful data, “Yeah, because if I can translate that question word by word and ask the participants and if I don’t understand myself the reason behind the question why we’re asking that, I might not get the response from this participant that the research is looking for.” (Participant 3). Community Researchers sometimes grapple with this responsibility, translating information across languages while staying true to the participant’s story and aims of the research, “Yeah, so you’re really juggling or balancing what the participant actually said to you, with what they mean, with how you can actually express that in a way that makes sense to an English-speaking person.” (Participant 8).

The experience remains inherently frustrating because there will always be understandings that are lost in translation, “… it’s always going to be like this because some words just don’t, what’s the word I’m trying to say, like it doesn’t translate to a particular language.” (Participant 4). The team has developed techniques for minimising these issues. We built a template for transcribing and translating interviews, creating space to explain, “So, I called my manager and I said, ‘Look these words I can’t say it in English. So, she said,’ That’s fine just make like a small detail what she is meaning, or like a small sentence”. Then she sent me a template for how to write the transcripts, how to interpret them to English. So, that was really helpful.” (Participant 1).

### 3.6. Finding the Right Way to Ask

Sensitive research topics can be understood differently across communities, “… cause the [name of community] people their background is like, how do you say, it’s oral learning, so have like academic questions, it’s hard to convey that because everyone has different learning style and how they understand things and how they work things, how they see the world.” (Participant 4). Community Researchers reflected on their experiences in interviews in ways that demonstrated their skills and ability to bring sensitive topics to life and ensure participants felt safe talking about challenging situations, “So, instead of just saying stillbirth, I have to find another word for just like this, you know, to explain what it means.” (Participant 8). The synthesis of lived experience, cultural and language expertise, and ongoing reflective practice facilitates the development of accessible language that makes sense to the community. For some communities, finding the right way to ask includes understanding the dynamic of community and language. Waves of migration over decades can change vocabulary and grammar, “We talk about language evolution, it changes over time.” (Participant 6). Community Researchers who work with these communities also need to understand how to choose the right form of language, depending on who they are talking to.

### 3.7. A Trauma-Informed Approach

Community Researchers bring a trauma-informed approach to practice, which emphasises establishing a physically, emotionally, and culturally safe space for participants and for Community Researchers, “… the bicultural officer [Community Researcher] is a lot more familiar with the community and how to go with them along the way.” (Participant 3); “I think it would be different if they had an interpreter and then someone else, like someone else interviewed them in English and some interpreter had to interpret.” (Participant 8); “… they might not understand why we are asking all these questions, why we are recording, and we need to be like sensitive that it can trigger trauma…” (Participant 4). The opportunity to attend relevant professional development was important, supporting the integration of trauma-informed principles to fieldwork, including the necessity of boundaries and self-care, “It felt empowering.” (Participant 4); “One thing that stand out to me was just I feel like that… self-care, because the stories that you hear, sometimes you know it’s not easy to hear…” (Participant 5).

A trauma-informed approach requires continuity to build trust and rapport, “I kind of call them up as many times as possible, I text them to see if they’re okay and if they’re okay with the dates for interview.” (Participant 8). Taking time to ensure participants are aware of their rights, “I explained about our policy, about how her information would be kept confidential. And I think that’s what made her to open up about it… a few of my participants raised issues about most of the time when they see like a counsellor, all this information, it was not shared with them.” (Participant 8). Maintaining awareness of factors that can impact on a person’s ability to participate, “… sometimes, when I was calling them, no answer. So that’s like I was understanding that I have to call later, or I was sending a message, like ‘text me and I’ll call you when I can’.” (Participant 2). Flexibility is necessary to gather the data, sometimes interviews are completed over several sessions or after hours if that is what a participant needs to tell their story, “It was like sometimes through the phone I had to do, continue an interview in three or four parts…” (Participant 2). Closure can be an important component of feeling heard and wherever possible Community Researchers keep participants up to date with study progress and outcomes, “I remember when I get back to them after the stories were written, they [the study participants] said, ‘You did a very good job, we don’t need to fix anything.’ So, I think that way it worked, it’s really good it’s working.” (Participant 1). Community Researchers also recommend that knowledge translation consistently includes the generation of translated resources that are accessible and useful for communities who have shared their stories and experiences.

### 3.8. Support within the Workplace

The right kind of support to facilitate this work means regular supervision to debrief, feel safe, and address challenges. “That gave us a power to do our work, like we feel there’s someone behind us, support us, helping us, and we have a hand any time, whenever.” (Participant 2). Access to meaningful professional development is necessary to feel competent and confident, “I think the team there was like really, like conscious of what skills would be important for us…” (Participant 6). Given the unpredictable nature of field work and what it takes from Community Researchers to bring a trauma-informed approach to their practice, supervisors and managers also need to remain flexible, “If she is not available, I just text her and she was really good at replying and fast.” (Participant 1); “I feel like they’re always there, if I needed something, I can always reach out to her and she would be, you know, respond back to me straight away. That was really helpful.” (Participant 8); “I just work at home whenever I’m available and the team are really flexible too which I like about it.” (Participant 3).

## 4. Discussion

Despite growing recognition of the importance of understanding the lived experience of culturally diverse populations [26,45], clinical and public health researchers have been slow to adapt research processes to facilitate participation for communities who are marginalised and ‘harder to reach’ [46,47]. In this study, we asked Community Researchers to reflect on the role they play in enabling meaningful participation in research for women, families, and communities of refugee and migrant background. The findings highlight the significant contributions made by Community Researchers at all stages of the research process. Community Researchers in this study were culturally and linguistically matched to the communities that they were seeking to engage. Knowledge of community and understanding of issues of concern were equally important to language skill in facilitating and conducting community engagement, recruitment, interviews, and discussion groups in culturally safe ways. Importantly, Community Researchers played a critical role in guiding the interpretation of study findings and ensuring that dissemination strategies appropriately represented issues of concern to families.

### 4.1. Community Researcher Expertise

Reflections from Community Researchers detail the processes required to engage with culturally diverse communities, demonstrating that facilitation of meaningful research participation requires the incorporation of cultural knowledge beyond interpretation and the use of translated research materials. As one Community Researcher stated, enabling community members to talk requires ‘building a bridge’ as a first step. Unfortunately, the important role Community Researchers hold as a cultural broker is under-recognised, and sometimes minimised or misrepresented in peer-reviewed literature [5,48,49,50,51]. While language and interpretation skills are vital in cross-cultural research, they constitute only one element of the knowledge and scope of Community Researcher expertise within our team [16].

When researchers in high income countries fail to recognise, value, or embrace the cultural knowledge and capacity of Community Researchers in facilitating meaningful participation for culturally diverse communities in research processes, there are inherent dangers that threaten the integrity of the data generated [46,52,53,54]. Ethically, it should not be acceptable for studies to exclude participants based on limited proficiency in the first languages of high-income countries [47]. Nor should it be acceptable for the engagement of communities and interpretation of lived experience to be solely undertaken by researchers who do not have community expertise, to ensure culturally specific and sensitive data collection and interpretation [54,55,56].

### 4.2. Integrating Community Researcher Expertise into Research Processes

Addressing the power dynamics that perpetuate the marginalisation or misrepresentation of culturally diverse communities in research requires an explicit commitment to valuing different ways of knowing and being [26,54]. Community Researchers in this study detailed multiple sources of knowledge that critically informed the development and conduct of the research they were involved in, including their lived experience as community members and people of refugee or migrant background. An iterative approach to data collection and analysis allowed the research team to learn together, grow understandings, and improve ways of working collaboratively and with a trauma-informed lens. Community Researchers themselves perceived that their ability to act as a bridge between research teams and communities is essential to meaningful engagement and reciprocal information exchange. Notably, Community Researchers identified two-way benefits for research teams and communities. Researchers can benefit from increased understanding of community priorities, and what communities need to feel confident about research processes or make informed decisions to participate. Communities can benefit from learning about health care in countries of settlement, what to expect when asked to participate in research, why research is relevant to them, their rights in health and research settings, and where to access additional support and information.

A detailed consideration of the challenges for research teams in integrating Community Researchers is beyond the scope of this paper. However, the authors are mindful that the approaches outlined in this paper take time and a genuine commitment to reflexive practice and to partnership with communities. The real costs of this and the importance of job security and career pathways for Community Researchers are rarely recognised by funding bodies. Secure employment for Community Researchers would enable research teams to provide greater opportunities for confidence and capacity building, career growth, study and leadership opportunities, and to increase cultural diversity and perspectives in the research workforce.

## 5. Conclusions

Our findings highlight the importance of an intentional, considered, and trauma-informed approach when conducting research with women, families, and communities of refugee and migrant background. Community Researchers culturally and linguistically matched to the background of those taking part in research have essential knowledge and skills to facilitate meaningful participation in research processes and strengthen data integrity and interpretation. Acknowledging Community Researcher expertise is a step toward recognising their vital contribution to culturally safe research practices, and the generation of perinatal research that is relevant and accessible for refugee and migrant background women, families, and communities. We recommend further research be undertaken to strengthen understanding of what it takes for Community Researchers to be the ‘cultural bridge’ between research and community groups, including the benefits and challenges inherent to the experience.

## Data Availability

Data not available due to the conditions of ethical approval. Participants were assured that raw data would not be shared.
